# Metyltetraprole, a novel putative complex III inhibitor, targets known QoI‐resistant strains of *Zymoseptoria tritici* and *Pyrenophora teres*

**DOI:** 10.1002/ps.5288

**Published:** 2019-01-17

**Authors:** Haruka Suemoto, Yuichi Matsuzaki, Fukumatsu Iwahashi

**Affiliations:** ^1^ Health & Crop Sciences Research Laboratory Sumitomo Chemical Co., Ltd Takarazuka Japan

**Keywords:** Metyltetraprole, *Zymoseptoria tritici*, *Pyrenophora teres*, QoI, resistance management

## Abstract

**BACKGROUND:**

Metyltetraprole is a new fungicide with a unique tetrazolinone‐moiety and a similar side chain to a known quinone outside inhibitor (QoI), pyraclostrobin. In this study we describe a unique bioactivity of metyltetraprole on QoI‐resistant strains of *Zymoseptoria tritici* and *Pyrenophora teres*.

**RESULTS:**

Metyltetraprole exhibited potent antifungal activity against Ascomycetes; it was especially effective against *Z. tritici* and P. teres in seedling pot tests. Metyltetraprole was also effective in field tests with QoI‐resistant mutants. Antifungal activity tests using field strains of *Z. tritici* and P. teres showed that the performance of metyltetraprole was unaltered by QoI, succinate dehydrogenase inhibitor (SDHI), and sterol 14α‐demethylation inhibitor (DMI) resistance. However, the mitochondrial activity test indicated that the compound inhibits the respiratory chain via complex III.

**CONCLUSION:**

Metyltetraprole is a novel fungicide that is highly effective against a wide range of fungal diseases, including important cereal diseases. Although metyltetraprole most likely inhibits the respiratory chain via complex III, it remains effective against QoI resistant strains. Therefore, metyltetraprole is considered as a novel fungicidal agent for controlling diseases affecting cereal crops and overcoming pathogen resistance to existing fungicides. © 2018 The Authors. *Pest Management Science* published by John Wiley & Sons Ltd on behalf of Society of Chemical Industry.

## 1 INTRODUCTION

Wheat and barley production in Europe is approximately 330 million metric tons per year, accounting for 40% of the global production (2016, FAOSTAT http://www.fao.org/faostat/en/#home). Septoria leaf blotch and net blotch are the most devastating foliar diseases in cereal crop production on this continent.^1^, ^2^, ^3^ The former, which is caused by *Zymoseptoria tritici*, can result in up to 50% of losses in yield in the case of severe epidemics,^4^ whereas barley production can be reduced by 10–40% by *Pyrenophora teres*, which is the causal agent of net blotch.^5^ At the same time, cereal crops are also vulnerable to several other diseases such as rust, tan spot, and ramularia leaf spot. As a countermeasure to this problem, considerable efforts have been made to breed new cultivars that are resistant to these key diseases. Nevertheless, not all pathogens can be controlled by this approach, therefore fungicides remain an important solution for protecting crops from diseases.

Agents for controlling foliar diseases on wheat and barley are typically applied two to four times during plant development.^6^, ^7^ Applying the agents around the time of flag leaf emergence is very important for preventing Septoria leaf blotch of wheat and net blotch of barley. However, the pathogens responsible for these diseases are evolving resistance towards either the sterol 14α‐demethylation inhibitors (DMIs) or the succinate dehydrogenase inhibitors (SDHIs), compromising the efficacy of the mixtures which have been widely used for cereal production.^8^

DMI resistance is associated with point mutations or overexpression of the enzyme encoded by *CYP51* and efflux pump overexpression.^9^, ^10^, ^11^, ^12^, ^13^ Resistance to SDHIs is also caused by mutations in the *Sdh* genes encoding the subunits of complex II in the respiratory chain.^14^, ^15^ The extent of reduction in sensitivity depends on the chemical structure of the fungicidal molecule, mutation type, and even the stacking of multiple types of mutations. DMI fungicides are losing efficacy against *Z. tritici* or *P. teres* 8 in regions of west Europe where these cereals are intensively produced. SDHIs are also reported to be under the risk of efficacy loss due to the field isolates which acquired a high level of resistance against this chemical class as a result of a single mutation within the target enzyme (e.g. *sdhC*‐H152R in *Z. tritici* and *sdhC*‐G79R in *P. teres*).^15^, ^16^

Similar observations have been made with regard to the quinone outside inhibitor (QoI) fungicides for which resistance rapidly developed after the introduction of first‐generation QoI fungicides.^17^ Although QoI fungicides have lost their efficacy against *Z. tritici* and *P. teres*, they are often used as a tank‐mix fungicide to control rust diseases or to achieve a greening effect. Because of this continuous exposure to QoI fungicides, the frequency of QoI resistance remains elevated in *Z. tritici* and *P. teres* populations. The QoI fungicides inhibit the complex III of the mitochondrial respiration chain. This enzyme transfers electrons through the redox reaction of ubiquinol and thus has binding sites for ubiquinone and ubiquinol (Qi and Qo sites, respectively). QoI fungicides bind to the Qo site and inhibit quinol oxidation, thereby blocking electron transfer.^18^, ^19^ Resistance to QoIs is caused by mutations in the *cyt‐b* gene encoding a component of mitochondrial respiratory chain complex III. Although there are a few mutation types that affect sensitivity to QoIs, the G143A mutation is the one conferring the highest level of resistance.^20^ This mutation predominates in *Z. tritici* resistant field populations^17^, ^21^, ^22^ and is assumed to possess almost no fitness disadvantage. Conversely, *P. teres* developed the widely spread F129L resistance mutation whereas the G143A substitution was never reported in this pathogen because the sequence coding for G143 is located immediately before an intron and its mutation leads to a lethal splicing error.^23^ The degree of sensitivity reduction associated with F129L varies according to the structure of QoI fungicide, whereas G143A confers high levels of resistance^24^ towards all existing QoI fungicides.

Given the increasing resistance of pathogens to commercially used fungicides, there is always a need for novel approaches to protect cereal crops from diseases. Resistance management can be achieved by identifying molecules with a novel mode of action (MoA) or by novel adoption of molecules from a known MoA group and previously never used in cereals or by modification of molecules not affected by cross‐resistance to existing analogs. The third approach could be achieved by adjusting chemical structures of molecules to improve interaction with mutated‐target sites.^25^, ^26^

**Figure 1:**
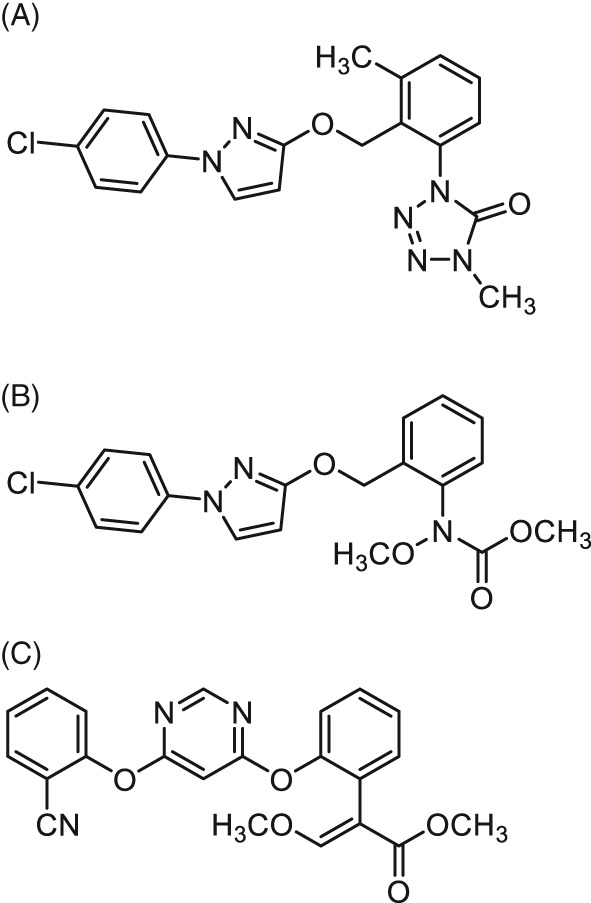
Structure of metyltetraprole and other major strobilurins: (a) metyltetraprole, (b) pyraclostrobin, (c) azoxystrobin.

In this study, we evaluated the efficacy of the novel chemical agent metyltetraprole in controlling Septoria leaf blotch and net blotch‐causing fungal strains that are resistant to the major fungicides. The chemical structure of metyltetraprole, 1‐(2‐{[1‐(4‐chlorophenyl)‐1*H*‐pyrazol‐3‐yl]oxymethyl}‐3‐methylphenyl)‐1,4‐dihydro‐4‐methyl‐5*H*‐tetrazol‐5‐one, is shown in Fig. 1. The side chain of metyltetraprole is similar to that of the QoI fungicide pyraclostrobin. Our investigation also suggested the mode of action of metyltetraprole is inhibition of the complex III but is not affected by QoI resistance mutations. Our results demonstrate that metyltetraprole is a promising agent for managing plant diseases caused by fungicide‐resistant pathogens.

## 2 MATERIALS AND METHODS

### 2.1 Chemical materials

For *in vitro* experiments, chemical compounds were dissolved in dimethyl sulfoxide (DMSO) as stock solutions. Metyltetraprole (99.1% purity) was synthesized by Sumitomo Chemical, Tokyo, Japan. Fluxapyroxad, pyraclostrobin, and prothioconazole‐desthio were purchased from Sigma‐Aldrich Japan, Tokyo, Japan. For efficacy tests on seedling pots or in fields, an emulsifiable concentrate (EC) formulation of metyltetraprole was prepared by Sumitomo Chemical. Pyraclostrobin (Comet, 200 g L^−1^ EC; BASF, Ludwigshafen am Rhein, Germany) was also used for greenhouse efficacy tests.

### 2.2 Plant materials

Seedlings of wheat (*Triticum aestivum* L. cv Apogee) and barley (*Hordeum vulgare* L. cv Nishinohoshi) were used for pot tests. Seedlings were grown to stage BBCH 12 in plastic pots (*n* = 5 plants per pot) in a growth room at 15 °C under a 14‐h day length.

### 2.3 Mycelial growth assay

The antifungal activity of metyltetraprole against *Z. tritici*, *Ramularia collo‐cygni*, *P. teres*, *Pyrenophora tritici‐repentis*, *Parastagonospora nodorum*, *Botrytis cinerea*, *Colletotrichum graminicola*, *Microdochium nivale*, *Rhizoctonia solani*, *Ustilago maydis*, *Aphanomyces cochlioides*, *Pythium irregulare*, and *Phytophthora capsici* was evaluated by two different methods under the incubation conditions detailed in Supporting Information Table S1.

#### 2.3.1 *96‐well microtiter plate method*

Growth of *Z. tritici*, *R. collo‐cygni*, *P. nodorum*, and *U. maydis* was evaluated on 96‐well microtiter plates. Conidia of *Z. tritici*, crushed mycelia of *R. collo‐cygni*, conidia of *P. nodorum*, or yeast‐like cell of *U. maydis* were harvested in distilled water and the density was adjusted with the appropriate medium (Supporting Information Table S1) to 1 × 10^4^ mL^−1^ conidia, crushed mycelia, or yeast‐like cell, respectively. A 100‐fold dilution series of metyltetraprole was prepared as a stock solution in DMSO and a 1‐µL aliquot was added to each well for a total of 11 test concentrations. A 99‐µL volume of prepared inoculum or medium without conidia/mycelia (blank) was added to each well. The final concentrations of metyltetraprole were 3, 1, 0.3, 0.1, 0.03, 0.01, 0.003, 0.001, 0.0003, 0.0001, and 0 mg L^−1^. The incubation conditions are shown in Supporting Information Table S1. Growth was measured by optical density at a wavelength of 600 nm with a microplate reader SH‐9000 Lab (Colona Electric, Ibaraki, Japan). Optical density values were corrected by the value for the blank well. The 50% effective concentration (EC_50_) was determined by probit analysis. One unit of experiment has four replicates of each concentration of metyltetraprole.

#### 2.3.2 *Agar plate method*

*P. teres*, *P. tritici‐repentis*, *B. cinerea*, *C. graminicola*, *M. nivale*, *R. solani*, *A. cochlioides*, *P. irregulare*, and *P. capsici* were cultured on agar media (see Supporting Information Table S1) amended with a series of concentrations of metyltetraprole (3, 1, 0.3, 0.1, 0.03, 0.01, 0.003, 0.001, 0.0003, 0.0001, and 0 mg L^−1^). Mycelium radial length was measured at designated days after inoculation and EC_50_ values were calculated. One unit of experiment has four replicates of each concentration of metyltetraprole.

#### 2.3.3 *Cross‐resistance test*

*Z. tritici* and *P. teres* strains used for the cross‐resistance test were isolated from infected leaves collected from the fields. Detached leaves were kept in humid conditions and spore formation was induced. A single spore was collected under the microscope and grown on potato dextrose agar (PDA) medium (39 g PDA in 1 L water). Sampling locations of isolates are listed in Supporting Information Table S2. Growth on fungicide‐containing medium was evaluated with the microtiter plate method (*Z. tritici*) or plated‐medium method (*P. teres*). Criteria for resistance were as follows: *Z. tritici* strains have EC_50_ > 1 mg L^−1^ against azoxystrobin (QoI),^27^ ≥0.5 mg L^−1^ against fluxapyroxad (SDHI),^16^ and >1 mg L^−1^ against bromuconazole (DMI).^28^ *P. teres* strains showed >20% growth in comparison to the untreated control on Yeast Bacto Acetate (YBA) medium plates containing 0.5 mg L^−1^ azoxystrobin, 5 mg L^−1^ boscalid (SDHI), and 1 mg L^−1^ bromuconazole. The tested concentrations of metyltetraprole, pyraclostrobin, fluxapyroxad, and prothioconazole‐desthio were 3, 1, 0.3, 0.1, 0.03, 0.01, 0.003, 0.001, 0.0003, 0.0001, and 0 mg L^−1^. The EC_50_ value was calculated based on the average inhibition rate of four replicates. Resistance factor (RF) was calculated using the formula RF = (EC_50_ of field isolate)/(EC_50_ of reference isolate).

### 2.4 Foliar spray tests on wheat

Plants were sprayed with diluted formulations at a spray volume of 200 L ha^−1^. Sporulation of *Zymoseptoria tritici* isolates was induced on malt yeast agar (10 g malt, 4 g yeast extract, 4 g glucose, 20 g agar, and up to 1 L with water) at 12 °C in the dark. Conidia were resuspended in water at 1 × 10^5^ mL^−1^. Prepared inoculum was sprayed onto the plant until the leaf surface was covered with a layer of fine droplets. Inoculation was performed 1 or 11 days after preventative or residual treatment, respectively. For curative treatment, inoculation was performed 5 days before fungicide application. Inoculated plants were kept in a growth chamber with a misting system for 3 days at 12 °C followed by incubation in a growth room at the same temperature under 14‐h light conditions. The plants were grown for about 3 weeks before the percentage of infected leaf area was evaluated. The efficacy of each treatment was calculated using the formula efficacy = 100 × [1 − (infected leaf area of treated plant/infected leaf area of untreated control)]. The assay was repeated twice with five replicates per treatment.

### 2.5 Foliar spray tests on barley

Plants were spray‐treated as described for wheat. *P. teres* sporulation was induced on double‐concentrated V8 juice medium (400 mL V8 vegetable juice, 3 g calcium carbonate, and 20 g agar, made up to 1 L with water) under a blacklight blue lamp. Prepared inoculum was sprayed onto barley seedlings. Inoculated plants were kept under humid conditions at 23–25 °C for 3 days and then transferred to normal greenhouse conditions at the same temperature. Infected leaves were detached and kept in saturated humid condition at 23–25 °C for 3–5 days to induce sporulation. The spores were harvested by washing with diluted water and the density was adjusted to 1 × 10^4^ mL^−1^ and used for inoculation of potted plants, which was performed 1 or 11 days after fungicide application for preventative or residual treatment. For curative treatment, plants were inoculated 2 days before fungicide application. Inoculated plants were incubated for 5–7 days before the percentage infected leaf area was assessed. The assay was repeated twice with five replicates per treatment.

### 2.6 Field trial

The field data presented in this report are based on 38 *Z. tritici* trials (13 in France, 9 in Germany, 8 in UK, 4 in Ireland, 3 in Italy, and 1 in Belgium) and 27 *P. teres* trials (13 in France, 2 in UK, 4 in Italy, 2 in Poland, and 1 each in Ireland, Austria, Hungary, Czech, Romania, and Bulgaria) conducted from 2015 to 2017. The trials were carried out by contractor companies according to the guidelines of the European and Mediterranean Plant Protection Organization (http://pp1.eppo.int/) of the year of study. The field efficacy of metyltetraprole at 120 g active ingredient ha^−1^ was tested, with pyraclostrobin (Comet; BASF) at 220 g active ingredient ha^−1^, prothioconazole (JOAO 250 g L^−1^ EC; Bayer CropScience, Monheim am Rhein, Germany), and fluxapyroxad (IMTREX 62.5 g L^−1^ EC; BASF) serving as reference treatments. The water volume was 200–250 L ha^−1^. All chemicals were applied with a hand‐held boom sprayer with conventional nozzles at T1 and T2 fungicide application timings. Disease severity was assessed and the percentage of disease control was calculated relative to the infection level of corresponding untreated leaves. Mean percentage of disease control was determined from the data of penultimate leaf at individual trials.

### 2.7 Preparation of submitochondrial fractions

Submitochondrial fractions of *Z. tritici* (Set 1 as a sensitive strain and Set 15‐2 as a resistance strain) and *P. teres* (Pt 6 as a sensitive strain and Pt 15‐1 as a resistance strain) were prepared as follows. *Z. tritici* and *P. teres* were grown to stationary phase (96 h) in 150 mL potato dextrose broth (PDB) at 23 °C. Hyphae were collected by centrifugation at 8000 × *g* for 15 min and resuspended in 40 mL of 20 mmol L^−1^ Tris–HCl (pH 7.5) containing 0.25 mol L^−1^ sucrose, 1% bovine serum albumin (BSA), and 2 mmol L^−1^ EDTA, and then homogenized with a French press at 4 °C. The homogenate was centrifuged at 1500 × *g* for 10 min; the supernatant was centrifuged at 15 000 × *g* for 20 min, and the membrane fraction was resuspended in 10 mL of 20 mmol L^−1^ Tris–HCl buffer (pH 7.5) containing 0.25 mol L^−1^ sucrose, 1% BSA, and 2 mmol L^−1^ EDTA. The suspension was centrifuged again at 15 000 × *g* for 20 min and the resultant precipitate was resuspended in 2 mL of mitochondria storage buffer (BioVision, Milpitas, CA, USA).

### 2.8 *In vitro* assay for mitochondrial electron transport activity

The succinate‐cytochrome c reductase (SCR) assay was carried out as previously described.^29^ Metyltetraprole and the other QoI fungicides were added to the SCR reaction mixtures as DMSO solutions. The final concentration of DMSO was 0.1%. The inhibitory activity of each fungicide was determined as the fungicide concentration required for 50% inhibition (IC_50_).

## 3 RESULTS

### 3.1 Characterization of metyltetraprole

Figure 1 shows the chemical structure of metyltetraprole. Metyltetraprole is characterized by its tetrazolinone moiety and the methyl group on the central bridging ring albeit it also has the same phenylpyrazole side chain as pyraclostrobin.

We first investigated the antifungal activity of metyltetraprole against *Z. tritici*, *R. collo‐cygni*, *P. teres*, *P. tritici‐repentis*, *P. nodorum*, *B. cinerea*, *C. graminicola*, *M. nivale*, *R. solani*, *U. maydis*, *A. cochlioides*, *P. irregulare*, and *P. capsici* (Table 1). The EC_50_ values of metyltetraprole against Ascomycetes ranged from 0.0020 to 0.054 mg L^−1^. Metyltetraprole showed especially potent growth inhibition of the fungi belonging to the orders Capnodiales and Pleosporales, and the class Sordariomycetes. The EC_50_ values against *Z. tritici* and *P. teres* were 0.0022 and 0.0048 mg L^−1^, respectively. On the other hand, the recorded EC_50_ values for Basidiomycetes and Oomycetes were higher than 0.040 and 0.82 mg L^−1^, respectively. These results demonstrate that metyltetraprole effectively suppresses the growth of Ascomycetes, while its activity against Basidiomycetes and Oomycetes was lower.

**Table 1:** Antifungal activity of metyltetraprole against various plant pathogens

Division	Class	Species	EC_50_ (mg L^−1^)^a^
Ascomycetes	Capnodiales	*Zymoseptoria tritici*	0.0022
		*Ramularia collo‐cygni*	0.0020
	Pleosporales	*Pyrenophora teres*	0.0048
		*Pyrenophora tritici‐repentis*	0.054
		*Parastagonospora nodorum*	0.0025
	Leotiomycetes	*Botrytis cinerea*	0.026
	Sordariomycetes	*Colletotrichum graminicola*	0.0068
		*Microdochium nivale*	0.0047
Basidiomycetes		*Rhizoctonia solani* AG2‐2 IIIB	>3.0
		*Rhizoctonia solani* AG4	2.2
		*Ustilago maydis*	0.040
Oomycetes		*Aphanomyces cochlioides*	0.82
		*Pythium irregulare*	>3.0
		*Phytophthora capsici*	>3.0

a Mean of four independent EC_50_ values.

### 3.2 Efficacy of metyltetraprole against *Z. tritici* on wheat seedlings

**Figure 2:**
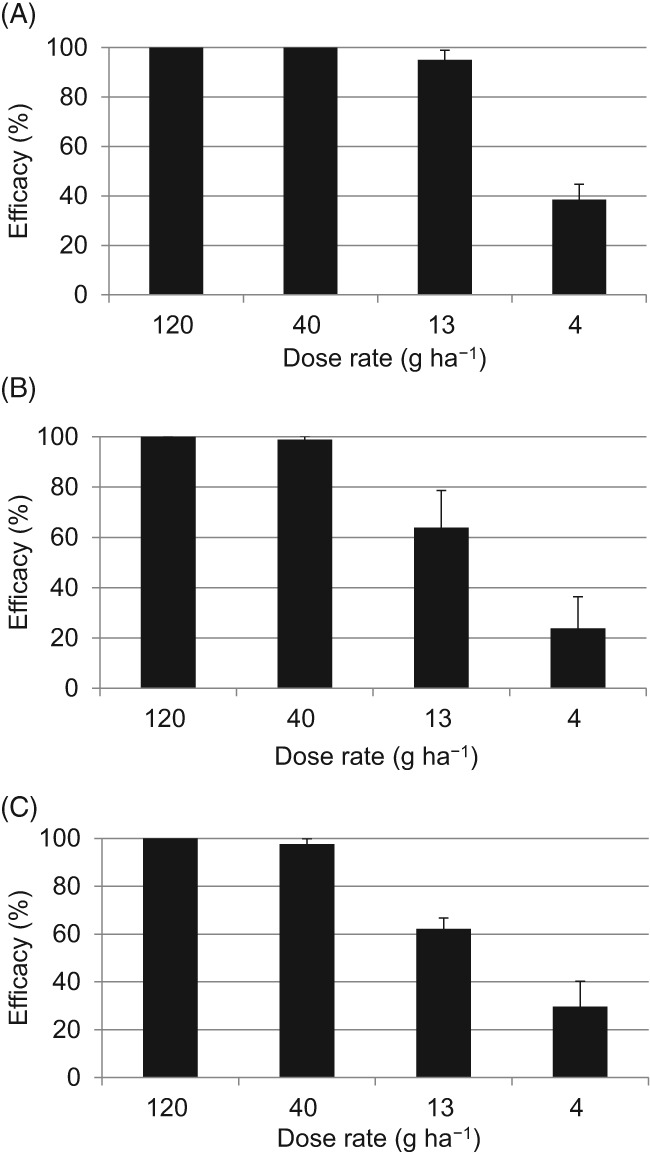
(A) Preventive, (B) residual, and (C) curative efficacy of metyltetraprole against Septoria leaf blotch on wheat seedlings. A sensitive strain of *Z. tritici* (Set 1) was inoculated. Disease severity in the untreated controls was 70% (A), 87% (B), and 93% (C). Error bars show SD.

We also evaluated the potential of metyltetraprole as a fungicide with the seedling pot test using *Z. tritici* (Fig. 2). Preventive application of metyltetraprole completely controlled *Z. tritici* infection at 120 and 40 g ha^−1^, respectively. The efficacies of 13 and 4 g ha^−1^ of metyltetraprole were 95.1% and 38.5%, respectively.

Metyltetraprole applied at 40 g ha^−1^ almost completely prevented Septoria leaf blotch, even when used for residual and curative treatment: for the former, the efficacy of 40 g ha^−1^ metyltetraprole was 98.8%, followed by 63.8% at 13 g ha^−1^ and 23.8% at 4 g ha^−1^, respectively. For curative treatment, the efficacy of 40 g ha^−1^ metyltetraprole was 97.6%, followed by 62.2% at 13 g ha^−1^ and 29.6% at 4 g ha^−1^, respectively. Thus, in seedling pot tests in a greenhouse, metyltetraprole effectively controlled Septoria leaf blotch.

### 3.3 Efficacy against P. teres in the pot test

**Figure 3:**
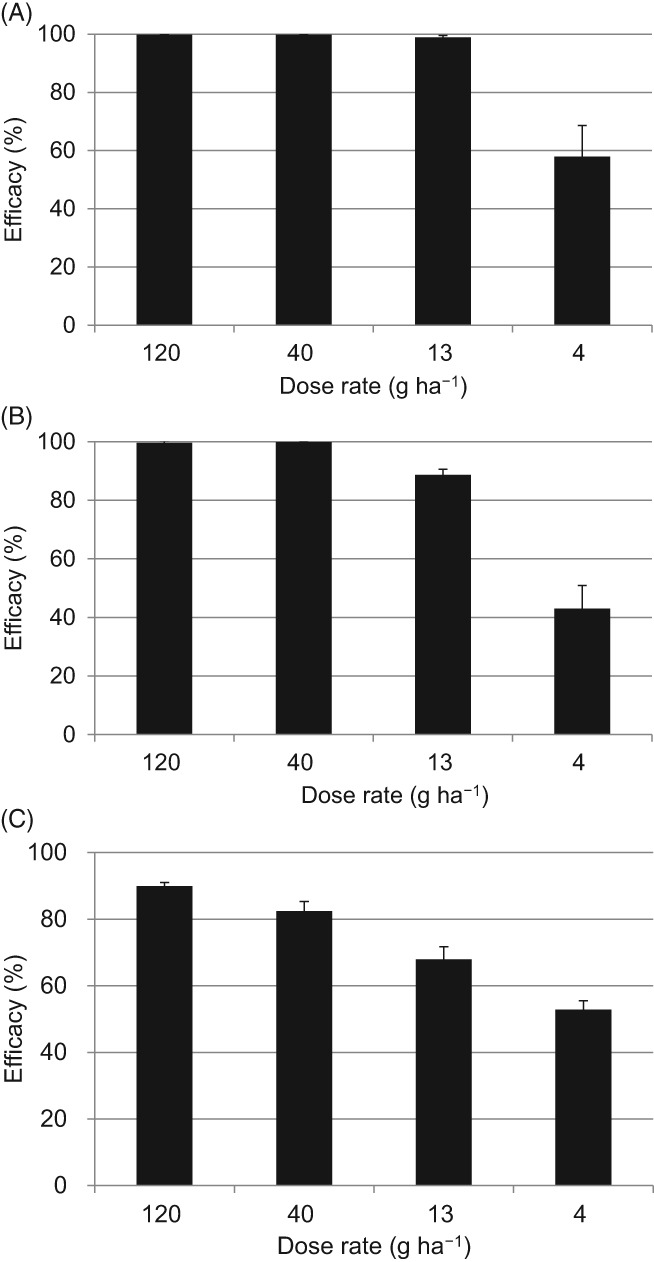
(A) Preventive, (B) residual, and (C) curative efficacy of metyltetraprole, against net blotch on barley seedlings. A sensitive strain of P. teres (Pt 6) was inoculated. Disease severity in the untreated control was 92% (A), 86% (B), and 77% (C). Error bars show SD.

We also investigated the ability of metyltetraprole to control *P. teres* infection when used as preventive, residual, and curative treatment (Fig. 3). Metyltetraprole showed almost full control at 120, 40, and 13 g ha^−1^ when applied as a preventative measure showing efficacy of 57.9% at 4 g ha^−1^. After 11 days of residual treatment, 120 and 40 g ha^−1^ metyltetraprole showed almost 100% control of infection, with efficacies of 88.7% at 13 g ha^−1^ and 43.0% at 4 g ha^−1^. Under curative conditions, 2 days after inoculation, the efficacy was reduced to 82.4% at 40 g ha^−1^ and 52.8% at 4 g ha^−1^. Thus, metyltetraprole can effectively prevent or mitigate net blotch on potted plants.

### 3.4 Field efficacy

The above results demonstrate that metyltetraprole can inhibit the growth of *Z. tritici* and *P. teres* on potted plants in a greenhouse. We also conducted field trials in Europe to evaluate the potential of metyltetraprole as an agricultural fungicide under field conditions, with three commercial agricultural fungicides (pyraclostrobin, prothioconazole, and fluxapyroxad) as references.

**Figure 4:**
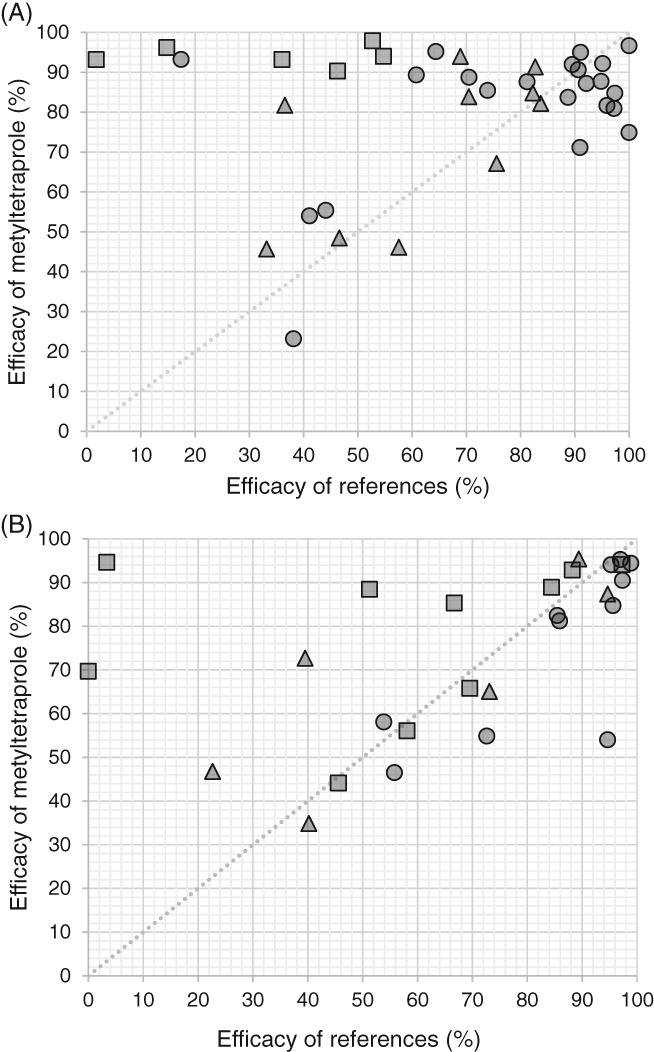
Field efficacy of metyltetraprole against (A) Septoria leaf blotch and (B) net blotch. Average disease pressure in untreated controls was (A) 54.1% and (B) 36.0%. Each plot shows the average efficacy in a single trial. The shape of the presented data points indicates the reference fungicide; squares are pyraclostrobin at 220 g ha^−1^, triangles are prothioconazole at 200 g ha^−1^, and circles are fluxapyroxad at 125 g ha^−1^. The dotted line is *y* = *x*, with the *y* and *x* axes showing the efficacy of metyltetraprole and of references, respectively.

In comparison with pyraclostrobin, the efficacy of metyltetraprole against septoria leaf blotch was high and stable (Fig. 4(A)). Based on the general information, we presumed the existence of resistant isolates to QoI fungicides and therefore conducted a sensitivity analysis of field isolates in 2015. We isolated five strains from each field and tested the antifungal activity of azoxystrobin with the microtiter plate method. Only 2/30 strains had an EC_50_ < 0.1 mg L^−1^, indicating that the resistant strains accounted for >90% of the population in the tested fields (Supporting Information Table S3). Metyltetraprole showed higher efficacy than pyraclostrobin against *Z. tritici*.

Metyltetraprole also showed a high, stable degree of efficacy against net blotch, in contrast to the variable efficacy of pyraclostrobin (Fig. 4(B)). Sensitivity tests on YBA plates amended with 0.5 mg L^−1^ of azoxystrobin were conducted in 2015; strains showing less than 20% growth compared to the untreated plate were categorized as sensitive strains. We conducted four trials in 2015 and found that pyraclostrobin showed lower efficacy in two fields (3.4% in trial 3 and 51.3% in trial 1 vs. 84.4% in trial 2 and 97.3% in trial 4). The percentage of resistant strains isolated from trials 2 and 4 was < 20%; however, 56% of isolates in trial 3 were resistant (Supporting Information Table S4). Since isolates from trial 1 were contaminated, the sensitivity data obtained from this trial were excluded. Nonetheless, our results indicate that the efficacy of pyraclostrobin was declined in fields with a higher proportion of strains of resistant biotypes whereas that of metyltetraprole was stable. Thus, metyltetraprole can control both *Z. tritici* and *P. teres* even in the presence of high proportions of QoI‐resistant strains in trial fields.

### 3.5 Seedling pot test using QoI‐resistant strain

**Figure 5:**
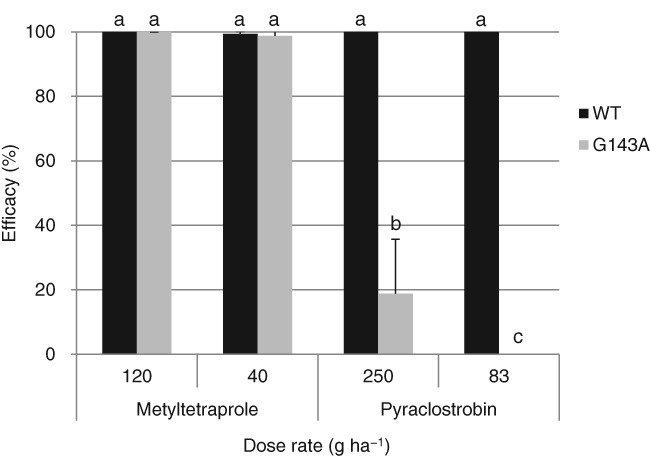
Efficacy of metyltetraprole and pyraclostrobin against *Z. tritici* isolates with and without *cyt‐b* mutation. WT, wild type (Set1); G143A, isolate with G143A mutation in *cyt‐b* (Set 15‐2). Error bars show SD. Different letters indicate significant differences between means at α = 0.05 (Tukey–Kramer test).

We evaluated the efficacy of metyltetraprole against the G143A mutant of *Z. tritici* under greenhouse conditions. Pyraclostrobin showed complete control of wild‐type strain at 83 g ha^−1^; however, the efficacy against the G143A mutant was significantly reduced (Fig. 5). In contrast, metyltetraprole controlled the G143A mutant to a degree comparable to the sensitive strain. These results indicate that the antifungal efficacy of metyltetraprole is unaffected by the presence of pyraclostrobin‐resistant strains.

### 3.6 Antifungal activity test against fungicide‐resistant strains

The antifungal activity of metyltetraprole was further assessed on field isolates of *Z. tritici* and *P. teres*, which showed low sensitivity to representative fungicides including pyraclostrobin (QoI), fluxapyroxad (SDHI), and prothioconazole (DMI) (Tables 2 and 3). Resistant field isolates were collected from various locations in Europe (Supporting Information Table S2).

**Table 2:** Antifungal activity of metyltetraprole, pyraclostrobin, fluxapyroxad, and prothioconazole‐desthio form against *Z. tritici* isolates with resistance to several groups of fungicide

	Sensitivity^a^			Metyltetraprole		Pyraclostrobin		Fluxapyroxad		Prothioconazole‐desthio	
Strain	QoI	SDHI	DMI	EC_50_ b	RF^c^	EC_50_	RF	EC_50_	RF	EC_50_	RF
Set 1	S	S	S	0.0038	—	0.0016	—	0.028	—	0.00089	—
Set 15‐1	R	S	R	0.0047	1.3	0.50	306.3	0.042	1.5	0.0045	5.0
Set 15‐2	R	S	R	0.0025	0.7	0.45	273.6	0.018	0.7	0.0072	8.1
Set 15‐3	R	R	R	0.0047	1.3	0.31	187.9	0.57	20.2	0.058	65.5
Set 15‐4	R	R	R	0.0055	1.5	0.37	225.8	0.50	18.0	0.019	21.3
Set 15‐5	S	S	R	0.0088	2.3	0.0016	1.0	0.021	0.75	0.021	23.4

a Sensitivity/resistance. R, resistant strain; S, sensitive strain.

b Mean of four independent EC_50_ values (mg L^−1^).

c Resistance factor is the ratio of EC_50_ of the resistant strain to that of the Set 1 (reference) strain.

**Table 3:** Antifungal activity of metyltetraprole, pyraclostrobin, fluxapyroxad, and prothioconazole‐desthio against isolates of P. teres with resistance to several groups of fungicide

	Sensitivity^a^			Metyltetraprole		Pyraclostrobin		Fluxapyroxad		Prothioconazole‐desthio	
Strain	QoI	SDHI	DMI	EC_50_ b	RF^c^	EC_50_	RF	EC_50_	RF	EC_50_	RF
Pt 6	S	S	S	0.007	—	0.002	—	0.009	—	0.007	—
Pt 15‐1	R	S	R	0.013	1.9	0.029	14.5	0.007	0.8	0.044	6.3
Pt 15‐2	S	R	R	0.015	2.1	0.002	1.0	0.748	83.1	0.067	9.6
Pt 15‐3	S	S	R	0.009	1.3	0.003	1.4	0.010	1.1	0.131	18.8
Pt 17‐1	R	S	R	0.011	1.6	0.033	16.5	0.011	1.2	0.073	10.4

a Sensitivity/resistance. R, resistant strain; S, sensitive strain.

b Mean of four independent EC_50_ values (mg L^−1^).

c Resistance factor is the ratio of EC_50_ of the resistant strain to that of the reference strain.

EC_50_ values of metyltetraprole against *Z. tritici* field strains ranged from 0.0025 to 0.0088 mg L^−1^ and were similar to that against a wild type strain Set1, which is sensitive to QoI, SDHI, and DMI fungicides. The RF (i.e. ratio of the EC_50_ value of a field isolates to that of Set 1) of metyltetraprole was <3. For example, Set 15‐3 was a triple‐resistant strain with RF values of 188 to pyraclostrobin, 20 to fluxapyroxad, and 66 to prothioconazole, although the EC_50_ of metyltetraprole against this strain was 0.0047 mg L^−1^. The RF value of 1.3 indicated that both Set 15‐3 and Set 1 were sensitive to metyltetraprole (Table 2).

*P. teres* strains resistant to representative fungicides were also sensitive to metyltetraprole (Table 3). Pt 15‐1 was resistant to pyraclostrobin and prothioconazole, with RF values of 14.5 and 6.3, respectively. Meanwhile, Pt 15‐2 was resistant to fluxapyroxad and prothioconazole, with RF values of 83.1 and 9.6, respectively. On the other hand, metyltetraprole showed similar antifungal activity levels against field strains and the sensitive strain Pt 6. The EC_50_ values of the other field strains were also similar to that of the wild‐type, that is, 0.007–0.015 mg L^−1^ and RF < 3. These data indicate that the activity of metyltetraprole is almost unaffected by the strains that show resistance to pyraclostrobin, fluxapyroxad, and prothioconazole.

### 3.7 Assay for electron transport activity in fungal respiration

To clarify the MoA of metyltetraprole, we examined its effect on the electron transport system of *Z. tritici* and *P. teres*. Succinate‐cytochrome c reductase (SCR) activity reflects electron transfer from succinate to cytochrome c via complex II and complex III. SCR activities were assayed by measuring the increase of the absorbance resulting from the reduction of cytochrome c.^30^ Metyltetraprole, azoxystrobin, and pyraclostrobin potently suppressed SCR activity in QoI‐susceptible *Z. tritici* and *P. teres*; the IC_50_ value of metyltetraprole against *Z. tritici* was 0.00025 mg L^−1^, which was smaller than that of azoxystrobin and similar to that of pyraclostrobin (Table 4). The IC_50_ value of metyltetraprole against *P. teres* was 0.0011 mg L^−1^, which was similar to that of azoxystrobin. Additionally, we tested the NADH dehydrogenase activity, which reflects electron transfer from NADH to complex III via complex I (complex I and III activities). Metyltetraprole and other QoI compounds inhibited the NADH dehydrogenase activity of fungal mitochondria (Supporting Information Table S5). Based on these observations and the chemical structure of metyltetraprole, we speculate that the target of metyltetraprole is mitochondrial complex III (i.e., ubiquinol‐cytochrome c reductase complex).

**Table 4:** Inhibitory activities of metyltetraprole, azoxystrobin, and pyraclostrobin against mitochondrial electron transport chain of *Zymoseptoria tritici* and *Pyrenophora teres*

A. *Z. tritici*							
		Metyltetraprole		Azoxystrobin		Pyraclostrobin	
Strain	Resistance mutation	IC_50_^a^	RF^b^	IC_50_	RF	IC_50_	RF
Set 1 (QoI‐S)	—	0.00025	—	0.0080	—	0.00026	—
Set 15‐2 (QoI‐R)	G143A	0.00042	1.7	1.92	240.0	0.20	769.2

B. *P. teres*							
		Metyltetraprole		Azoxystrobin		Pyraclostrobin	
Strain	Resistance mutation	IC_50_ a	RF^b^	IC_50_	RF	IC_50_	RF
Pt 6 (QoI‐S)	—	0.0011	—	0.0041	—	0.00044	—
Pt 15‐1 (QoI‐R)	F129L	0.0058	5.4	0.25	60.9	0.016	36.1

a Mean of three independent IC_50_ values (mg L^−1^).

b Resistance factor is the ratio of the IC_50_ of the QoI‐resistant (QoI‐R) strain to that of the QoI‐sensitive (QoI‐S) strain.

Metyltetraprole potently inhibited SCR activity in Set 15‐2 and Pt 15‐1, which are QoI‐resistant strains of *Z. tritici* and *P. teres*, respectively*,* albeit with very minor sensitivity shifts compared to their respective wild types (RF of 1.7 and 5.4, respectively) and compared to current QoI fungicides azoxystrobin and pyraclostrobin (RF of azoxystrobin = 240.0 and 60.9, respectively and RF of pyraclostrobin = 769.2 and 36.1, respectively).

## 4 DISCUSSION

Metyltetraprole is a new fungicide that has broad‐spectrum activity against Ascomycetes and is especially effective against Capnodiales, Pleosporales, and Sordariomycetes, which are fungal orders responsible for several important cereal diseases. Metyltetraprole showed potent antifungal activity against *Z. tritici*, *R. collo‐cygni*, *P. teres*, and *P. tritici‐repentis* (Table 1). The antifungal activity of metyltetraprole against *Z. tritici* and *P. teres* was comparable to that of pyraclostrobin, fluxapyroxad, and prothioconazole (Tables 2 and 3), three widely used fungicides, as demonstrated also in field tests (Fig. 4). Metyltetraprole also effectively controlled *R. collo‐cygni* and *P. tritici‐repentis* in the field (data not shown).

QoI fungicides bind to the Qo site of cytochrome b to inhibit complex III of the respiratory chain.^17^, ^31^, ^32^, ^33^ We speculated that metyltetraprole inhibits complex III due to its structural similarity to the other QoI fungicides (Fig. 1); this was supported by the results of the enzymatic activity assay (Table 4). Interestingly, the antifungal activity test revealed that metyltetraprole remains effective against the strains resistant to pyraclostrobin, which inhibits the same target, complex III. Under greenhouse conditions, metyltetraprole showed similar efficacy against the strains harboring the G143A substitution and sensitive strains, whereas the efficacy of pyraclostrobin against the resistant strains was significantly decreased (Fig. 5). Metyltetraprole also showed significantly higher efficacy than pyraclostrobin in the field where QoI‐resistant strains were detected at a high percentage (Fig. 4, Supporting Information Tables S3 and 4). The ratios of QoI‐resistant strains were 93% for *Z. tritici* and 24% for *P. teres*. This is consistent with a previous work demonstrating that all *Z. tritici* isolates sampled in the UK in 2015 harbored the G143A^22^ and that 24% of *P. teres* obtained in France in 2005 had F129L.^23^ The results of the field trial indicate that metyltetraprole can control diseases in cereal crops even in the field where a high prevalence of QoI‐resistant strains is expected.

G143A and F129L substitutions in cytochrome b affect the binding of QoI fungicides to the Qo site of the cytochrome bc1 complex, thereby reducing fungicidal efficacy.^18^ Analysis of the co‐crystal structure of cytochrome b with azoxystrobin revealed that the G143A narrows the pocket and thus alters the binding of azoxystrobin.^19^ On the other hand, the F129L interferes with the main interaction between the binding site and the pharmacophore of the fungicide.^19^ QoI fungicides were inspired by natural fungicidal derivatives such as strobilurin A and oudemansin A. Because natural strobilurins are readily degraded when exposed to UV, commercial QoI fungicides have been modified from strobilurin A to have greater stability so that they can remain effective for a few weeks in the field.^31^, ^32^, ^33^ But each of the existing QoI fungicides, which have been used as cereal fungicides, possesses a characteristic ‘strobilurin‐like’ structure as its pharmacophore, methoxyacrylate group, or methoxyacrylate‐like structure. Compared with pyraclostrobin and other commercial QoI, metyltetraprole has a unique tetrazolinone structure with a neighboring methyl group in a central bridging ring. These structural features may alter the interaction of metyltetraprole with the target binding pocket, which possesses G143A or F129L substitution. Therefore, the activity of metyltetraprole is not reduced by G143A and F129L. This hypothesis is supported by our molecular simulation between metyltetraprole and fungal complex III. It was revealed that the tetrazolinone moiety of metyltetraprole having a narrow range of partial charges and a spherical and compact shape might be effective against resistant mutant fungi (under submission). We will continue the study of the MoA of metyltetraprole.

Efficient crop production is essential for feeding the global population, especially with food shortages caused by climate change becoming a major problem.^34^ However, intensive use of agrochemicals has accelerated the development of resistance to a point where some pesticides are no longer effective.^35^ It is important to devise a sustainable approach to crop protection that integrates different methods. For example, biological control of agricultural pests has been intensively researched. Nevertheless, conventional chemical pesticides are still indispensable, especially for stable production of cereals in regions where several devastating diseases exist due to climatic conditions. Adoption of mixtures or rotation of pesticides with a distinct MoA is often recommended to avoid the development of resistance.^36^, ^37^, ^38^ However, the number of known distinct MoAs for fungicides is limited. Approximately 80% of the commercially used fungicides belong to only six MoA groups.^39^ Furthermore, the success rate in discovering novel targets for fungicide is declining year by year owing to difficulties arising from stronger regulatory restrictions.^40^ As an alternative strategy, several fungicides have been developed without exhibiting cross‐resistance with fungicides which have the same MoA. For example, some newer SDHIs (ex. fluopyram and isofetamid) were reported to show no cross‐resistance with boscalid, an older SDHI fungicide, on some isolates with specific mutation.^25^, ^26^ QoI is also one of the most important classes of fungicides owing to its broad spectrum and effectiveness in disease control. However, up to now, a novel analog of QoI fungicides, which overcomes cross‐resistance with existing QoI fungicides, has not been reported, especially for the mutants with G143A substitution.

In this study, we reported the unique profile of metyltetraprole, showing stable antifungal activity and efficacy in both the greenhouse and field against QoI‐resistant disease pathogens while targeting the same Qo site. We believe metyltetraprole is the first molecule which overcomes the cross‐resistance among QoI fungicides in the disease management of cereal production, therefore it can be used as a new tool for the sustainable management of crop pathogens. We also expect that further novel compounds with a similar tetrazolinone structure can be discovered as new types of highly effective agricultural fungicides. Our findings contribute to ongoing efforts to minimize losses of economically important crops through improved management of relevant diseases.

## Supporting information


**File S1.**
