# Supplementary material for: Metyltetraprole, a novel putative complex III inhibitor, targets known QoI‐resistant strains of Zymoseptoria tritici and Pyrenophora teres
Source: Pest Manag Sci. 2019 Jan 17;75(4):1181–9. doi: 10.1002/ps.5288 (PMC6590148; doi:10.1002/ps.5288)
Supplement: Supplementary file 1 — File S1. [file PS-75-1181-s001.doc]

**Supporting Information**

**Supporting Information 1.** References for medium preparation

**Supporting Information 2.** Method of *in vitro* assay for mitochondrial NADH dehydrogenase activity

**Supplemental Reference**

**Table S1.** Incubation conditions for antifungal activity tests

**Table S2.** Sampled country and year of European Union field isolates used for cross-resistance test

**Table S3.** Ratio of resistant strains of *Z. tritici* against azoxystrobin among isolates sampled from efficacy test fields

**Table S4.** Ratio of resistant strains of *P. teres* against azoxystrobin among isolates sampled from efficacy test fields

**Table S5.** Inhibitory activities of metyltetraprole, azoxystrobin, and pyraclostrobin against NADH dehydrogenase of *Z. tritici* and *P. teres*

**Supplemental Reference**

**Supporting information**

**Supporting Information 1.** References for medium preparation

Malt yeast agar (MYA) and yeast bacto glycerol medium (YBG):

Stammler G, SEPTTR microtiter monitoring method BASF 2009 V1, 2009, <http://www.frac.info/docs/default-source/monitoring-methods/approved-methods/septtr-microtiter-monitoring-method-basf-2009-v1.pdf?sfvrsn=ad99419a_4> [accessed 9 July 2018]

Alkyl ester broth (AEB):

Rohel EA, Cavelier N and Hollomon DW, Microscopic analysis of the effect of azoxystrobin treatments on *Mycosphaerella graminicola* infection using green fluorescent protein (GFP)-expressing transformants, *Pest Manag Sci* **57**:1017–1022 (2001).

V8 juice medium (V8):

Sierotzki H and Frey R, SEPTTR monitoring method Syngenta 2006 V1, 2006, <http://www.frac.info/docs/default-source/monitoring-methods/approved-methods/septtr-monitoring-method-syngenta-2006-v1.pdf?sfvrsn=a899419a_4> [accessed 9 July 2018]

**Supporting Information 2.** Method of *in vitro* assay for mitochondrial NADH dehydrogenase activity

The NADH dehydrogenase assay was carried out as previously described.^41^ Metyltetraprole and the other QoI fungicides were added to the NADH dehydrogenase reaction mixtures as DMSO solutions. The final concentration of DMSO was 0.1%. The inhibitory activity of each fungicide was determined as the fungicide concentration required for 50% inhibition (IC_50_).

**Supplemental Reference**

41 Long J, Ma J, Luo C, Mo X, Sun L, Zang W, et al., Comparison of two methods for assaying complex I activity in mitochondria isolated from rat liver, brain and heart, Life Sci 85:276–280, Elsevier Inc. (2009).

**Table S1.** Incubation conditions for antifungal activity tests

| **Fungal name** | **Medium**^a^ | **Incubation temperature (°C)** | **Incubation period (days)** | **Pre-incubation for sporulation** | **Test method** |
| --- | --- | --- | --- | --- | --- |
| ***Zymoseptoria tritici*** | YBG | 12 | 5 | 12°C for 7days on MYA | Microtiter plate |
| ***Ramularia collo-cygni*** | AEB | 18 | 7 | − | Microtiter plate |
| ***Pyrenophora teres*** | YBA | 23 | 7 | − | Plated medium |
| ***Pyrenophora tritici-repentis*** | PDA + tebfenpyrad 1 mg L^−1^ | 18 | 7 | − | Plated medium |
| ***Parastagonospora nodorum*** | YBA | 18 | 7 | 12°C for 7 days on V8 with BLB light | Microtiter plate |
| ***Botrytis cinerea*** | PDA + SHAM 100 mg L^−1^ | 18 | 4 | − | Plated medium |
| ***Colletotrichum graminicola*** | PDA + SHAM 100 mg L^−1^ | 23 | 7 | − | Plated medium |
| ***Michrodochium nivale*** | PDA + SHAM 100 mg L^−1^ | 18 | 4 | − | Plated medium |
| ***Rhizoctonia solani* AG2-2 IIIB** | PDA + SHAM 100 mg L^−1^ | 23 | 4 | − | Plated medium |
| ***Rhizoctonia solani* AG4** | PDA + SHAM 100 mg L^−1^ | 23 | 4 | − | Plated medium |
| ***Ustilago maydis*** | PDB | 18 | 4 | − | Microtiter plate |
| ***Aphanomyces cochlioides*** | PDA | 23 | 4 | − | Plated medium |
| ***Pythium irregulare*** | PDA + SHAM 100 mg L^−1^ | 18 | 3 | − | Plated medium |
| ***Phytophthora capsici*** | PDA + SHAM 100 mg L^−1^ | 23 | 7 | − | Plated medium |

^a^ AEB, alkyl ester broth; BLB, blacklight blue; MYA, malt yeast agar; PDA, potato dextrose agar; PDB, potato dextrose broth; V8, V8 juice medium; YBA, yeast bacto acetate medium (20 g yeast extract, 20 g peptone, 40 g sodium acetate, up to 1 L with water); YBG, yeast bacto glycerol medium (10 g yeast extract, 10 g peptone, 20 ml glycerol, up to 1 L with water)

**Table S2.** Sampled country and year of European Union field isolates used for cross-resistance test

| **Strain** | **Sampled country** | **Sampled year** |
| --- | --- | --- |
| Set 15-1 | France | 2015 |
| Set 15-2 | France | 2015 |
| Set 15-3 | UK | 2015 |
| Set 15-4 | Ireland | 2015 |
| Set 15-5 | France | 2015 |
| Pt 15-1 | Germany | 2015 |
| Pt 15-2 | France | 2015 |
| Pt 15-3 | France | 2015 |
| Pt 17-1 | Poland | 2017 |

**Table S3.** Ratio of resistant strains of *Z. tritici* against azoxystrobin among isolates sampled from efficacy test fields

| **Trial no.** | **Country** | **Number of isolates** | **Number of resistant strains**^a^ | **Percentage of resistant strains (%)** | **Efficacy of Metyltetraprole (%)** | **Efficacy of Pyraclostrobin**  **(%)** |
| --- | --- | --- | --- | --- | --- | --- |
| 1 | France | 5 | 4 | 80 | 93.2 | 36.0 |
| 2 | France | 5 | 5 | 100 | 96.2 | 14.8 |
| 3 | Germany | 5 | 4 | 80 | 93.2 | 1.8 |
| 4 | UK | 5 | 5 | 100 | 90.3 | 46.3 |
| 5 | UK | 5 | 5 | 100 | 94.0 | 54.7 |
| 6 | Ireland | 5 | 5 | 100 | 97.9 | 52.7 |
| Total | − | 30 | 28 | 93 | − | − |

^a^ Strains have EC_50_ > 1 mg L^−1^ against azoxystrobin.

**Table S4.** Ratio of resistant strains of *P. teres* against azoxystrobin among isolates sampled from efficacy test fields

| **Trial no.** | **Country** | **Number of isolates** | **Number of resistant strains**^a^ | **Percentage of resistant strain (%)** | **Efficacy of Metyltetraprole (%)** | **Efficacy of Pyraclostrobin**  **(%)** |
| --- | --- | --- | --- | --- | --- | --- |
| 1 | France | − | − | − | 88.4 | 51.3 |
| 2 | France | 16 | 2 | 13 | 88.9 | 84.4 |
| 3 | France | 9 | 5 | 56 | 94.6 | 3.4 |
| 4 | France | 12 | 2 | 17 | 94.1 | 97.3 |
| Total | − | 37 | 9 | 24 | − | − |

^a^ Strains showed > 20% growth in comparison to the untreated control on YBA medium plates containing 0.5 mg L^−1^ azoxystrobin.

**Table S5.** Inhibitory activities of metyltetraprole, azoxystrobin, and pyraclostrobin against NADH dehydrogenase of *Z. tritici* and *P. teres*

A. *Z. tritici*

| **Strain** | **Resistance mutation** |  | **Metyltetraprole** | |  | **Azoxystrobin** | |  | **Pyraclostrobin** | |
| --- | --- | --- | --- | --- | --- | --- | --- | --- | --- | --- |
|  |  |  |  |  |  |  |  |  |  |  |
|  |  |  | IC_50_^a^ | RF^b^ |  | IC_50_ | RF |  | IC_50_ | RF |
| Set1 (QoI-S) | − |  | 0.00028 | − |  | 0.0044 | − |  | 0.00022 | − |
| Set 15-2 (QoI-R) | G143A |  | 0.0012 | 4.4 |  | 2.58 | 584.3 |  | 0.11 | 486.3 |

*B.* P. teres

| **Strain** | **Resistance mutation** |  | **Metyltetraprole** | |  | **Azoxystrobin** | |  | **Pyraclostrobin** | |
| --- | --- | --- | --- | --- | --- | --- | --- | --- | --- | --- |
|  |  |  |  |  |  |  |  |  |  |  |
|  |  |  | IC_50_^a^ | RF^b^ |  | IC_50_ | RF |  | IC_50_ | RF |
| Pt 6 (QoI-S) | − |  | 0.0013 | − |  | 0.0065 | − |  | 0.00029 | − |
| Pt 15-1 (QoI-R) | F129L |  | 0.0061 | 4.7 |  | 0.34 | 52.3 |  | 0.018 | 62.1 |

^a^ Mean of three independent IC_50_ values (mg L^−1^).

^b^ Resistance factor is the ratio of the IC_50_ of the QoI-resistant (QoI-R) strain to that of the QoI-sensitive (QoI-S) strain.
